# Peritoneal carcinomatosis index predicts survival in colorectal patients undergoing HIPEC using oxaliplatin: a retrospective single-arm cohort study

**DOI:** 10.1186/s12957-019-1618-4

**Published:** 2019-05-15

**Authors:** Atuhani Burnett, Marie-Eve Aubé Lecompte, Nora Trabulsi, Pierre Dubé, Mai-Kim Gervais, Bertrand Trilling, Alexis-Simon Cloutier, Lucas Sideris

**Affiliations:** 1 Centre de Recherche, Hôpital Maisonneuve-Rosemont, Université de Montréal, Montréal, QC Canada; 2 Banner MD Anderson Cancer Center, Greeley, 80631 CO USA

**Keywords:** Colorectal cancer, HIPEC, Oxaliplatin, Peritoneal carcinomatosis

## Abstract

**Abstract:**

**Background:**

Peritoneal carcinomatosis (PC) from colorectal cancer is associated with poor prognosis. Cytoreductive surgery (CRS) combined with hyperthermic intraperitoneal chemotherapy (HIPEC) has improved survival for patients with colorectal peritoneal carcinomatosis. However, standardization of HIPEC protocols, including which chemotherapeutic agent to use, is lacking in the literature. Therefore, we sought to report survival outcomes from colorectal cancer patients undergoing CRS/oxaliplatin-based HIPEC at our institution over the last 10 years.

**Methods:**

Colorectal PC patients treated with CRS/oxaliplatin-based HIPEC 2004–2015 were included. Demographic, clinical, and oncologic data were abstracted from the medical record. Overall (OS) and disease-free survival (DFS) were calculated using Kaplan–Meier analysis. Univariate/multivariate Cox regression analysis was done.

**Results:**

Laparotomy was performed in 113 patients for colorectal PC; 91 completed a curative intent CRS/HIPEC. At 3 and 5 years, OS for the CRS/HIPEC cohort was 75% and 55%, and DFS was 50% and 25%, respectively. On multivariate analysis, incremental increases in peritoneal carcinomatosis index (PCI) were associated with worse OS (*p* = 0.0001) and DFS (*p* = 0.0001). Grade III/IV complications were also associated with worse OS.

**Conclusions:**

A standardized regimen of CRS and oxaliplatin-based HIPEC for colorectal PC is effective with favorable OS and DFS and acceptable complication rates.

## Background

Peritoneal carcinomatosis (PC) affects up to 30% of colorectal cancer patients and remains the second leading cause of death after liver metastasis [1, 2]. PC treated with palliative chemotherapy alone is associated with a median survival of 5.2–12.6 months [3]. However, in the early 1990s, complete cytoreductive surgery (CRS) combined with hyperthermic intra-peritoneal chemotherapy (HIPEC) was introduced by Dr. Sugarbaker as a new treatment for isolated colorectal and appendiceal PC [4]. Few randomized trials are available studying CRS/HIPEC; however, Verwaal et al. showed in their landmark randomized trial from the Netherlands that colorectal PC treated with CRS/HIPEC using mitomycin C had a median survival of 22.2 months compared to 12.6 months when treated with chemotherapy alone [5, 6]. Multiple retrospective studies, such as Elias et al., subsequently demonstrated an improved 5-year overall survival of 51% when PC was treated with CRS/HIPEC compared with 13% when these patients were treated with chemotherapy alone [3]. As a result, a curative intent complete cytoreduction combined with HIPEC is now considered the standard of care for PC of colorectal origin with an expected long-term survival of 40% at 5 years [7].

The cytoreductive surgery has been increasingly standardized over the years with complete removal of macroscopic carcinomatosis achieved through radical organ resection, omentectomy, peritoneal stripping, and ablation of any disease that cannot be resected [8]. Contraindications to proceeding with cytoreduction include extensive and unresectable mesenteric, small bowel, or hepatoduodenal involvement [9]. The peritoneal carcinomatosis index score is also now universally used to evaluate extent of disease, communicate between clinicians and investigators, and has been found to be prognostic regarding patient survival.

Unfortunately, despite the consensus on the importance of CRS/HIPEC in the management of colorectal PC, there has not been consensus on the optimal HIPEC approach, standardization of the protocol, nor on the best chemotherapeutic agent to use during the procedure. HIPEC can be delivered with the abdomen open [5, 6] or with the skin temporarily closed using large bore drains and a pump for chemotherapy circulation [9, 10]. Intra-peritoneal chemotherapy can be given at room temperature, post-operatively, using intra-abdominal drains (EPIC) [11, 12], instead of the heated (42–43 °C) intra-operative chemotherapy given during HIPEC [11, 13]. Some protocols call for IV chemotherapy to be infused at the time of HIPEC, [14] while others do not [5, 6]. Several different chemotherapeutic agents have been used for intraperitoneal chemotherapy including mitomycin C [5, 6, 10, 15], cisplatin [16–18], oxaliplatin [14].

Given the wide heterogeneity of methodology and approaches to CRS/HIPEC in both clinical practice and the literature, there is great benefit from conducting a study to assess the survival benefit in colorectal PC patients in the setting of a homogeneous protocol with a homogeneous chemotherapeutic agent. Therefore, we present here perioperative and oncologic outcomes from a cohort of colorectal PC patients presenting to a large Canadian referral center. The primary outcomes of overall survival and disease-free survival compare favorably with the literature, and secondary endpoints including complications were also acceptably low. On multivariate analysis, we confirmed the well-known prognostic value of PCI in predicting long term OS and DFS.

## Methods

### Patients and pre-operative assessment

All patients treated for PC with CRS and HIPEC at the Maisonneuve Rosemont Hospital (University of Montréal, Canada) between 2004–2015 were prospectively recorded in the institutional electronic medical record (EMR) and research database. This study was approved by the scientific committee of the Maisonneuve Rosemont Hospital, and all patients received the standard of care. Patients were assessed for eligibility for surgery using the Canadian HIPEC guidelines [19]. Exclusion criteria included disease progression during neoadjuvant chemotherapy, extra-abdominal disease, involvement of retroperitoneal lymph nodes, multiple hepatic metastases, or severe comorbidities. Appendiceal adenocarcinoma patients were not included in this cohort. Diagnosis was confirmed through histologic review and tumor markers. Pre-operative evaluation included computed tomography scan (CT) and positron emission tomography (PET-CT). Diagnostic laparoscopy was performed prior to HIPEC at a separate time to rule out unresectable disease in cases concerning for extensive miliary disease, pelvis frozen by carcinomatosis, extensive small bowel, or mesenteric disease.

### Systemic chemotherapy and radiotherapy

All patients received 6 months of neo-adjuvant FOLinic acid, Fluorouracil, OXaliplatin (FOLFOX) or FOLinic acid, Fluorouracil, IRInotecan (FOLFIRI) with or without bevacizumab to reduce the tumor burden and maximize the possibility of achieving complete cytoreduction. Bevacizumab was stopped at least 6 weeks prior to surgery. All patients with rectal cancer also received neo-adjuvant radiotherapy.

### Cytoreduction and HIPEC

A midline laparotomy incision followed by extensive adhesiolysis of the abdominal cavity was performed in each case. The extent of disease was assessed using the peritoneal carcinomatosis index (PCI) score [8]. Based on the PCI score and an assessment of complete resectability, an intra-operative decision to perform cytoreduction was made. Patients who were deemed to be unresectable were closed and sent for additional chemotherapy. All visible disease was removed through resection of affected organs, resection of carcinomatosis deposits, fulguration of smaller carcinomatosis nodules, and omentectomy to achieve a complete cytoreduction score of 0 (CCR0) or 1 (CCR1, all remaining disease is less than 2.5 mm in size). Bowel anastomoses were performed after the HIPEC until 2008, when anastomoses were performed before the HIPEC. The open approach for HIPEC [14, 20] was performed until 2011, when all HIPECs were performed using the closed approach [21, 22]. Details of our HIPEC procedure has been previously described [14]; however, in brief, patients receive a systemic dose of fluorouracil (5-FU) (400 mg/m^2^) and leucovorin (20 mg/m^2^) 1 hour prior to HIPEC. After cytoreduction has been completed, drains and temperature probes are placed, the abdomen closed, and heated D5W solution is circulated until temperature reaches 40 °C. Oxaliplatin is then infused (460 mg/m^2^) and a target temperature of 41–42 °C is maintained during the 30 min recirculation. If a patient had neuropathy from pre-operative FOLFOX, the HIPEC oxaliplatin dose was reduced to 300 mg/m^2^.

### Follow-up

Patients were seen in clinic, with a CEA level and a CT scan of the chest, abdomen, and pelvis, every 4 months for the first 2 years, every 6 months years 2–5, and yearly thereafter. A PET-CT was used alternately with CT or in case of concerning findings on the CT scan.

### Data collection and endpoints

Data collected for each patient are demographics, primary cancer, surgical procedure, complications (Clavien Dindo) [19], prognostic factors, length of hospitalization, follow-up, recurrence, and death. We considered a post-operative period of 60 days for the evaluation of peri-operative mortality and morbidity.

The primary endpoints were OS and DFS. Secondary endpoints were prognostic factors associated with survival and major complications.

### Statistical methods

Simple descriptive statistics were used to report patient, disease, and peri-operative events characteristics. Overall and disease-free survival estimates were calculated based on the initial presentation of carcinomatosis and demonstrated using Kaplan–Meier survival curves with log-rank tests to compare different subgroups. Univariate and multivariate Cox regression analysis was used to assess the relation between different factors and the occurrence of death or recurrence. These covariates included age, sex, primary tumor location, histology, grade and stage, peri-operative use of chemotherapy, synchronous vs metachronous presentation, PCI, operative duration, estimated blood loss, post-operative complications, and oxaliplatin dose. A *p* value of ≤ 0.05 was considered significant. Violation of proportional hazard assumption was assessed using the scaled Schoenfeld residuals method. Statistical analyses were performed using RStudio (version 0.99.893—© 2009–2016 RStudio, Inc.).

## Results

**Fig. 1:**
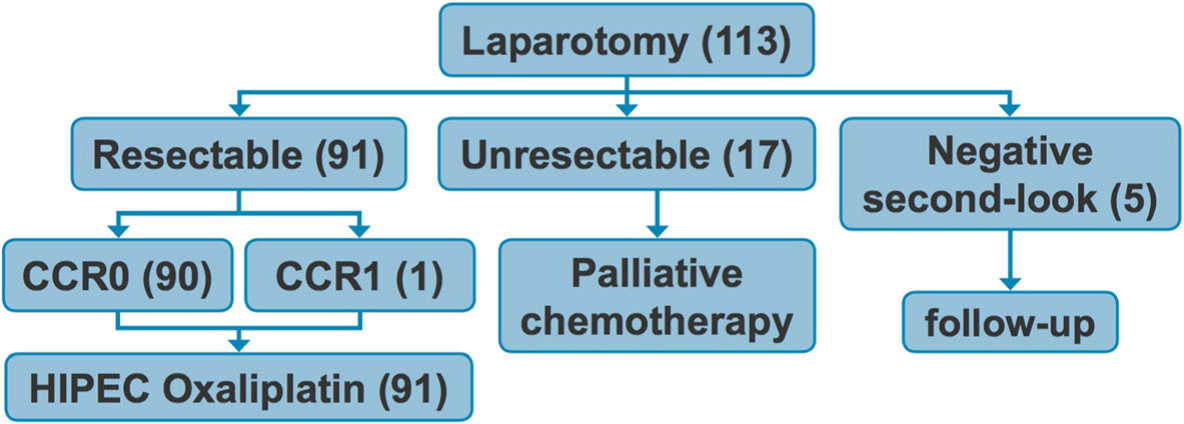
Schematic of study design

Of the 113 PC patients who underwent laparotomy at our center, 91 were resectable and underwent CRS/HIPEC. Ninety patients achieved a CCR score of 0, while one patient had a CCR score of 1. Twenty-two patients were considered unresectable during exploration at our center, were closed without cytoreduction, and went on to receive more systemic chemotherapy. Five of those patients were brought back for a second-look laparotomy, but all remained unresectable and were not cytoreduced (Fig. 1, schematic; Table 1).

**Table 1:** Demographics

	*N*
All patients	113
PC status	
Resectable	91
Unresectable	17
Unresectable—neg second look	5
Sex	
Male	59
Female	54
Age	
Median (range)	55 (33–71)
ASA	
I	6
II	77
III	29
IV	1
Presentation of PC	
Synchronous primary resected	45
Synchronous primary unresected	8
Metachronous	60
Primary tumor	
Colon	102
Rectum	11
Grade	
I	22
II	57
III	34
Stage on initial presentation	
I	1
II	17
III	38
IV M	4
IV PC	53
Pathology	
Intestinal adenocarcinoma	92
Mucinous adenocarcinoma	21

Patient demographics are described in Table 1 for the entire cohort (*N* = 113). Median age was 55 years (range 33–71), with 59 males (52%) and 55 females. Median ASA score was II. Median follow-up was 24 months (range 0–137). Most tumors were cancers of the colon (*N* = 102, 90%), with fewer rectal tumors (*N* = 11, 10%). Histological distribution was classical intestinal adenocarcinoma (*N* = 92, 81.4%) and mucinous adenocarcinoma (*N* = 21, 18.6%) (Table 1).

Fifty-three (46.9%) patients from our entire cohort presented with synchronous stage IV peritoneal disease. Most of these patients had been previously explored at other centers, given systemic chemotherapy, and referred to our center. Regarding their initial exploration, 17 patients had only the primary tumor resected, 28 had the primary resected with an incomplete cytoreduction, 4 patients were explored without any resection, and 4 patients had a complete cytoreduction and HIPEC at initial exploration at our center (Table 1).

The remaining 60 (53.1%) patients presented with metachronous peritoneal disease. Four patients (3.5%) presented initially with stage IV disease in other metastatic locations, 38 (33.6%) with stage III disease, 17 (15.0%) with stage II disease, and 1 (0.9%) patient with stage I disease (Table 1).

For the patients who underwent CRS/HIPEC, 81 were colon cancer as the primary site and 10 were of rectal origin (Table 2). The median PCI was 6 (range 0–23) with 5 patients having PCI > 20. A complete cytoreduction (CCR0) was achieved in all but one case (CCR1). A median of 2 organs were resected (range 0–6), and a median of 1 (range 0–3) anastomosis was performed in each case. Median operative time was 330 min (range 105–690 min), and blood loss was 375 cc (100–5000 ml). Most patients were admitted post-operatively to the ICU (*N* = 76, 83.5%) and required parenteral nutrition (*N* = 68, 74.7%). Median hospitalization was 16 days (range 6–67). Sixty-day post-operative morbidities and mortalities are listed in Table 2. There were a total of 10 patients (11.0%) with severe grade III/IV complications, and 1 mortality (1.1%) within the post-operative period (Table 2).

**Table 2:** Intra-operative and post-operative characteristics

	*N*	Percent	Median	Range
All patients (HIPEC)	91	100		
Primary tumor				
Colon	81	89		
Rectum	10	11		
CC score				
0	90	98.9		
1	1	1.1		
2	0	0		
PCI			6	0–23
HIPEC oxaliplatin dose				
300 mg/m^2^	12	13.2		
460 mg/m^2^	79	86.8		
Organ resected			2	0–6
Anastomosis			1	0–3
Operative time (min)			330	105–690
Open vs close abdomen				
Open	29	32		
Close	62	68		
IV Chemotherapy				
Yes (5-FU + LV)	68	75		
No	23	25		
Blood loss (ml)			375	100–5000
Blood transfusion	4	4.4		
Admission to ICU	76	83.5		
Parenteral nutrition	68	74.7		
Length of stay (days)			16	6–67
Post-operative complications				
None	43	47.2		
Grade 1	26	28.6		
Grade 2	11	12.1		
Grade 3	5	5.5		
Grade 4	5	5.5		
Grade 5	1	1.1		
Follow-up (months)			24	0–137

### Survival

**Fig. 2:**
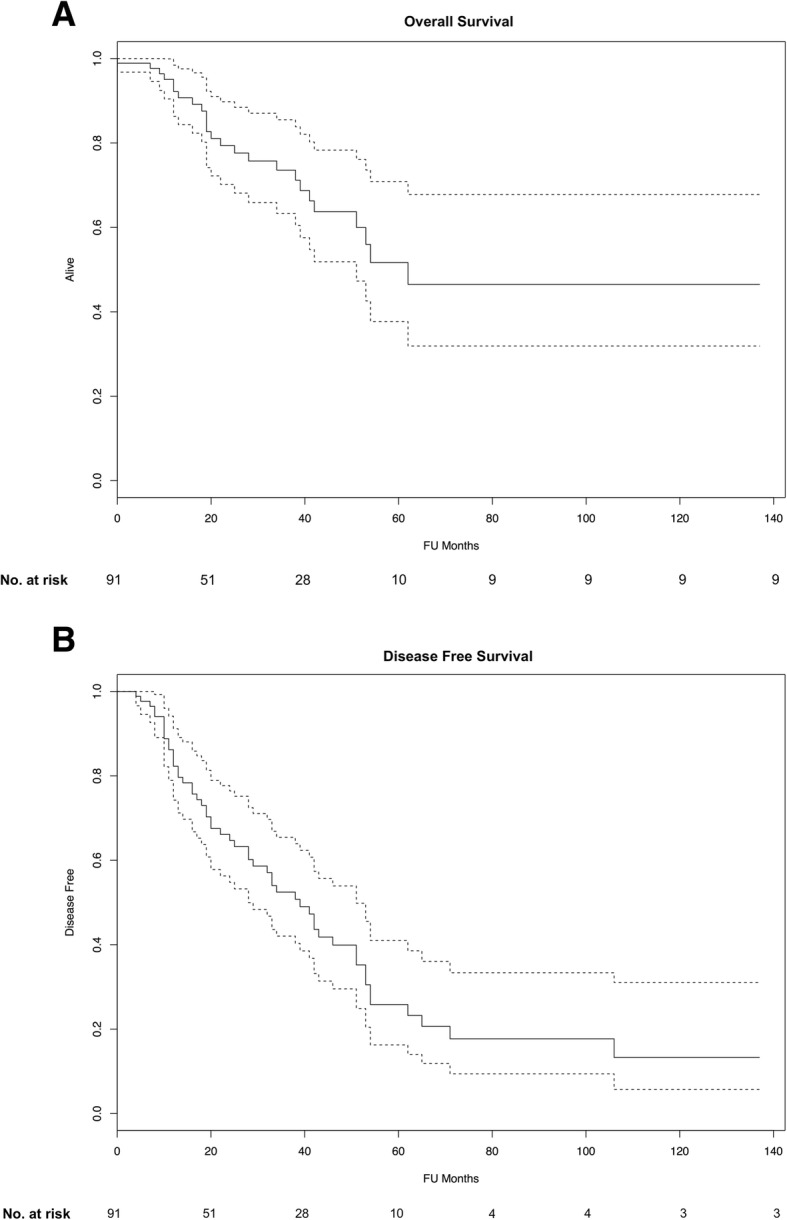
Kaplan–Meier survival analysis of **a** overall survival (OS) and **b** disease-free survival (DFS) for the entire cohort. Dotted lines represent 95% confidence interval

For the 91 patients who underwent CRS/HIPEC, OS at 3 and 5 years was 75% and 55%, with a median OS of 63 months (Fig. 2a). DFS at 3 and 5 years was 50% and 25%, with a median DFS of 36 months (Fig. 2b).

Predictors of overall survival were analyzed by univariate and multivariate analysis (Table 3). On univariate analysis, worse overall survival was found to be associated with every incremental increase in PCI (HR 1.134/PCI, *p* < 0.001); longer operative times (HR 1.198, *p* = 0.0355); greater blood loss (HR 1.039/100 ml, *p* = 0.0263), and grade III/IV complications (HR 7.242, *p* = 0.0012; HR 6.852, *p* = 0.003). When these parameters were further analyzed by multivariate analysis, PCI (HR 1.178/PCI, *p* = 0.0001) remained as a predictor of poor OS, along with grade III/IV complications (HR 6.324, *p* = 0.0208; HR 5.588, *p* = 0.0238). No association was found for age, gender, location of primary tumor, tumor histology, grade, presenting stage, synchronous vs metachronous peritoneal presentation, peri-operative chemotherapy, intra-operative chemotherapy (IV 5-FU/leucovorin), and grade I–II complications.

**Table 3:** Univariate and multivariate analysis for overall survival

Covariates	Univariate analysis			Multivariate analysis		
	HR	95% CI	*p* value	HR	95% CI	*p* value
Age	0.991	(0.951–1.032)	0.659			
Female	Ref					
Male	2.152	(0.974–4.751)	0.058			
Colon primary	Ref					
Rectal primary	1.268	(0.436–3.689)	0.663			
Classic adenocarcinoma	Ref					
Mucinous adenocarcinoma	1.037	(0.415–2.589)	0.938			
Grade I (histology)	Ref					
Grade II (histology)	0.845	(0.331–2.156)	0.725			
Grade III (histology)	0.889	(0.309–2.562)	0.828			
Presenting stage I–III	Ref					
Presenting stage IV	1.116	(0.367–3.395)	0.846			
Metachronous	Ref					
Synchronous	1.028	(0.471–2.245)	0.945			
Peri-op chemo	0.5350	(0.159–1.8)	0.788			
Intra-op IV chemo	0.8957	(0.402–1.997)	0.697			
PCI (continuous)	1.134	(1.074–1.198)	**< 0.001**	1.178	(1.083–1.281)	**0.0001**
Operative time (hours)	1.198	(1.012–1.417)	**0.0355**	0.772	(0.5493–1.086)	0.1375
Blood loss (100 ml)	1.039	(1.005–1.076)	**0.0263**	1.004	(0.947–1.064)	0.889
No complications	Ref					
Grade I (complication)	2.0827	(0.704–6.247)	0.187	2.173	(0.713–6.624)	0.172
Grade II (complication)	1.692	(0.422–6.775)	0.457	3.292	(0.749–14.48)	0.114
Grade III (complication)	7.242	(2.229–24.73)	**0.0012**	6.324	(1.383–28.919)	**0.0174**
Grade IV (complication)	6.852	(1.905–52.42)	**0.003**	5.588	(1.256–24.865)	**0.0238**

Boldface font indicate statistically significant *p* values of less than 0.05

The same covariates were analyzed for predictors of poor disease-free survival (Table 4). Again, incremental increases in PCI was found to predict poor DFS on both univariate (HR 1.101/PCI, *p* = 1.09e−06) and multivariate (HR 1.100/PCI, *p* = 0.0001) analysis. Peri-operative chemo was found to predict improved DFS on the univariate analysis (HR 0.419, *p* = 0.034) but not on the multivariate analysis. Similarly, operative time was found to predict poor DFS on univariate analysis (HR 1.199, *p* = 0.002) but not on the multivariate analysis. All other covariates were negative for association.

**Table 4:** Univariate and multivariate analysis for disease free survival

Covariates	Univariate analysis			Multivariate analysis		
	HR	95% CI	*p* value	HR	95% CI	*p* value
Age	1.026	(0.995–1.059)	0.096			
Female	Ref					
Male	1.4503	(0.8437–2.493)	0.179			
Colon primary	Ref					
Rectal primary	0.7183	(0.2847–1.812)	0.484			
Classic adenocarcinoma	Ref					
Mucinous adenocarcinoma	1.1205	(0.5979–2.1)	0.723			
Grade I (histology)	Ref					
Grade II (histology)	0.90843	(0.4726–1.746)	0.773			
Grade III (histology)	0.73676	(0.3409–1.592)	0.437			
Presenting stage I–III	Ref					
Presenting stage IV	1.4028	(0.8112–2.426)	0.226			
Metachronous	Ref					
Synchronous	1.2770	(0.7426–2.196)	0.377			
Peri-op chemo	0.419	(0.1873–0.938)	**0.034**	0.448	(0.1968–1.022)	0.056
Intra-op IV chemo	1.7762	(0.9555–3.302)	0.069			
PCI (continuous)	1.101	(1.059–1.145)	**1.09e−06**	1.100	(1.047–1.156)	**0.0001**
Operative time (hours)	1.199	(1.068–1.346)	**0.002**	1.014	(0.875–1.175)	0.856
Blood loss (100 ml)	1.0177	(0.9875–1.047)	0.265			
No complications	Ref					
Grade I (complication)	1.072	(0.510–1.996)	0.838			
Grade II (complication)	1.105	(0.472–2.576)	0.817			
Grade III (complication)	1.936	(0.739–5.208)	0.185			
Grade IV (complication)	1.849	(0.616–1.795)	0.260			

Boldface font indicate statistically significant *p* values of less than 0.05

**Fig. 3:**
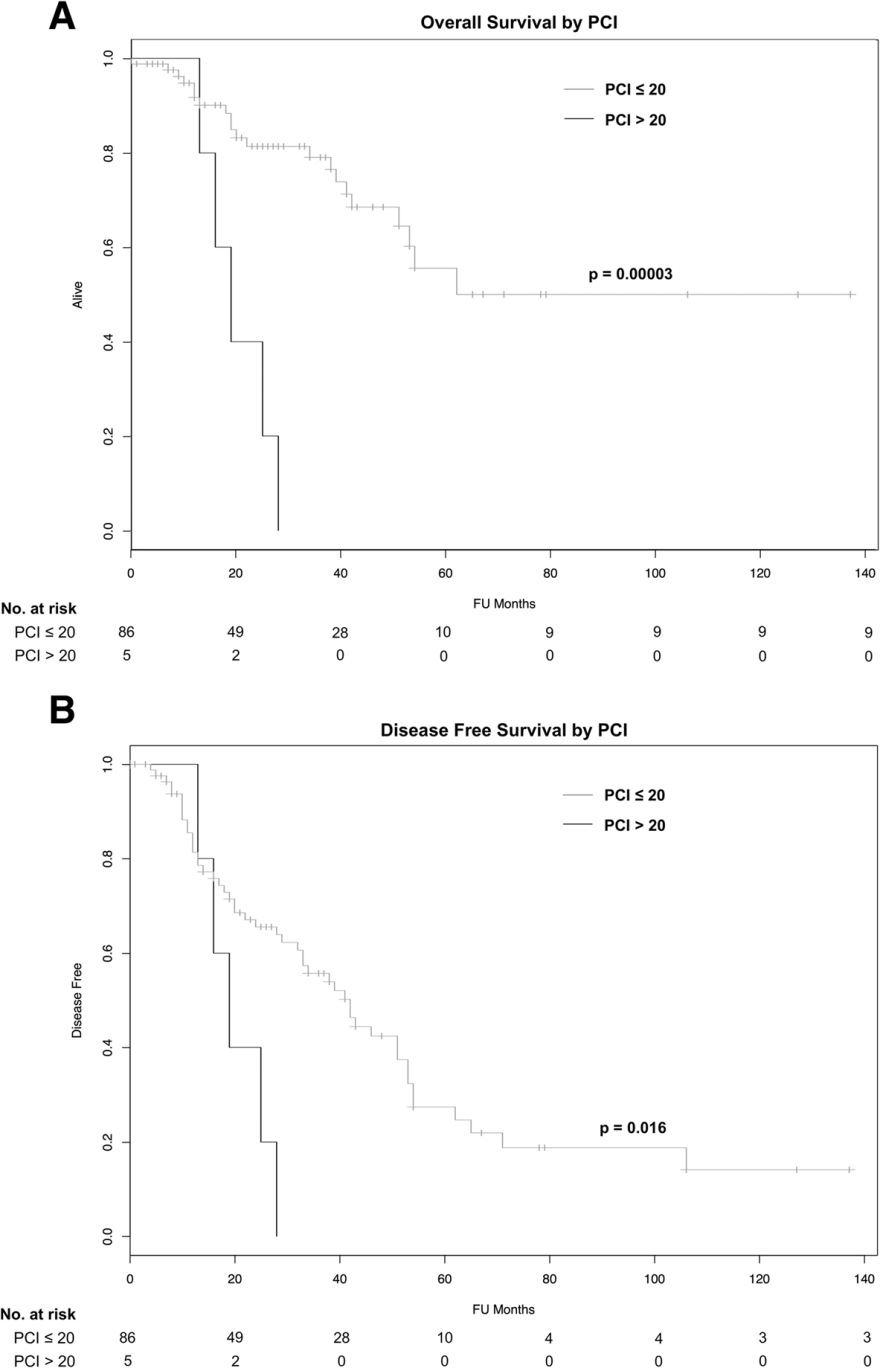
Kaplan–Meier survival analysis of **a** overall survival stratified by PCI of 20 and **b** disease-free survival stratified by PCI of 20

Patients were stratified by those having a PCI greater than 20 and those whose PCI was 20 or less. Median overall survival for patients having PCI > 20 was 19 months, while median OS for patients having PCI ≤ 20 was 62 months, which was statistically significant (Fig. 3a, *p* = 0.00003). Median DFS for patients with PCI > 20 was 19 months, while median DFS for patients having PCI ≤ 20 was 42 months, which was statistically significant (Fig. 3b, *p* = 0.016).

## Discussion

Peritoneal carcinomatosis arising from colorectal origin was once considered a terminal stage IV condition but now is represented by a spectrum of disease severity amenable to curative resection in the context of systemic and peritoneal chemotherapy. While CRS and HIPEC have come to be regarded as the standard of care, significant progress still needs to be made in standardizing the approach and chemotherapeutic agents used. One of the reasons for this lack of consensus is the lack of high-quality studies with homogeneous disease, techniques, and chemotherapeutic agents. Consequently, our study fills an important need, providing a relatively homogeneous population of colorectal carcinomatosis patients, all managed with systemic chemotherapy, CRS, and HIPEC with oxaliplatin. Additionally, patients were managed by the same surgical group at a tertiary referral center, using the same inclusion criteria developed in the Canadian HIPEC guidelines [19]. Our study follows up and expands on the patient population originally reported on by our group in 2013 [14]. This earlier study reported 40 patients who underwent CRS and oxaliplatin HIPEC with a 5-year survival of 33%. Our present study has added to this overall population for a total of 91 patients who underwent CRS and oxaliplatin HIPEC with a 5-year survival of 55%, grade III–IV complication rate of 11.0%. Finally, our study confirms that oxaliplatin-based HIPEC retains PCI as a relevant prognostic factor that can be used as a continuous variable in evaluating patients for surgery.

Our OS of 55% exceeds the rates quoted in the literature of 32–47% [23] and several factors could account for this. First, our center has a strict patient selection policy based on the Canadian HIPEC guidelines created jointly by our institution and others in Canada. This provides us with a more selected patient population. Secondly, the completeness of cytoreduction (CCR) score is known to be a powerful prognostic indicator in carcinomatosis patients [13, 24]. In our patients, however, we were able to achieve a CCR score of 0 in 99% of patients, which would naturally improve the OS of our entire cohort compared with studies where the CCR scores were more heterogeneous [11]. Third, PCI is known to be a significant prognostic factor, and our guidelines recommend against performing HIPEC in PCI > 20. Therefore, our patient population is biased toward lower PCI (with a median of 6) and this could contribute to the improved OS. Lastly, most HIPECs performed in the USA use mitomycin C as the chemotherapeutic agent [11, 24]; therefore, one hypothesis is that oxaliplatin is simply a more effective HIPEC agent than mitomycin C. However, this hypothesis would need to be tested in a head-to-head randomized trial comparing the two agents to be validated.

Although mitomycyin C is quite widely used as the agent of choice in the USA, our center has been using oxaliplatin as the primary agent for our protocol for several years due to a number of reasons [3]. First, there is a significant amount of moderate-quality evidence ranging from pharmacokinetics, animal studies, single institution retrospective, and few randomized trials that multiple agents including mitomycin C, cisplatin, oxaliplatin, and even heated water are effective during HIPEC. Secondly, oxaliplatin has a long proven track record of being active against colorectal tumors as an IV drug and the backbone of FOLFOX-based regimens. Lastly, pharmacokinetics studies in animals and humans indicate that oxaliplatin has a favorable tissue penetration profile (392 ng oxaliplatin/mg peritoneal tissue) [25–27].

The peritoneal carcinomatosis index (PCI) has been well-documented as an indicator of prognosis in patients receiving CRS + HIPEC in the setting of several agents including oxaliplatin [7, 8, 11, 13, 14]. However, there has been controversy on the most appropriate PCI cutoff value to use as an exclusion criteria when evaluating patients for CRS + HIPEC. Several studies, including the current Canadian HIPEC guidelines, recommend excluding colorectal carcinomatosis patients with a PCI > 20 [11, 19]; however, PCI values of 14 [13] and 16 [7] have been proposed by others. By expanding the original patient population reported by our group, we were able to stratify the population by PCI as a continuous variable. Our multivariate analysis was able to show a statistically significant reduction in both OS and DFS with each incremental increase in the PCI. The practical implications of this are that even when a particular PCI value is chosen as a clinical exclusion criterion, this should be considered only as a relative contraindication for young, healthy patients with favorable tumor biology who may still benefit from aggressive surgical intervention. Therefore, using the example of the five patients with PCI > 20 in our series, they achieved a median survival of 19 months which was inferior to the 62 months achieved in patients with PCI < 20 (Fig. 3a). However, these patients exceeded the historical expectation of 6–12-month overall survival for all comers treated with palliative chemotherapy and therefore may have been provided a benefit by undergoing CRS and HIPEC. This is of course acknowledging that our CRS HIPEC patient population has already been selected to be healthy and young enough to undergo the procedure and therefore might be more likely to have a longer (12 months) survival with palliative chemotherapy than the average stage IV patient with carcinomatosis.

Our study has limitations relating to the retrospective design, potential for clinician bias without blinding, referral bias, and the lack of a control arm for comparison. Nevertheless, we have attempted to control for these factors in a number of ways. First, the data was collected prospectively and all patients that presented were included in the study. We cannot mitigate the referral bias; however, we would expect this to worsen our survival outcomes due to receiving more complex patients. Lastly, we referred to historical controls as our reference population both for patients treated with palliative chemotherapy and patients receiving comparable CRS/HIPEC under different regimens which we felt were acceptable given the large body of literature describing both of these populations.

## Conclusions

Taken together, we feel that our study advances the understanding of the precise effectiveness and survival advantages of an oxaliplatin-based CRS and HIPEC in a homogeneous population of colorectal carcinomatosis patients. Our study also conclusively re-emphasizes the importance of PCI in prognosis and patient selection even in a cohort of patients achieving excellent CCR0 disease control. Finally, given the favorable survival outcomes and low morbidity profile we have reported, our protocol can serve as a template for oxaliplatin-based HIPEC programs.

## Abbreviations

PC: Peritoneal carcinomatosis
CRS: Cytoreductive surgery
HIPEC: Hyperthermic intraperitoneal chemotherapy
OS: Overall
DFS: Disease-free survival
PCI: Peritoneal carcinomatosis index
EPIC: Early post-operative intra-peritoneal chemotherapy
EMR: Electronic medical record
CT: Computed tomography
PET-CT: Positron emission tomography
FOLFOX: FOLinic acid, Fluorouracil, OXaliplatin
FOLFIRI: FOLinic acid, Fluorouracil, IRInotecan
CCR: Completeness of cytoreduction
5-FU: Fluorouracil
D5W: 5% dextrose in water
ICU: Intensive care unit
